# Agile meeting method for building intelligent decision support systems

**DOI:** 10.1016/j.mex.2023.102311

**Published:** 2023-08-02

**Authors:** Guilherme Nascimento Pate Santos, Carlos José Pereira de Lucena

**Affiliations:** Computer Science Department of Pontifical Catholic, University of Rio de Janeiro, Marquês de São Vicente Street, 225 - CEP, Rio de Janeiro 22453-900, Brazil

**Keywords:** Software engineering, Agile method, Data enrichment, Conceptual framework, Method for Intelligent Decision Support System, Agile Meeting Method

## Abstract

In this article, we present an agile method based on a cycle of meetings that guides the construction of intelligent decision support systems. This method presents the phases of initiation, analysis and planning, negotiation, control and intelligent decision support. A cycle represents a passage through all the phases of the method, where as the execution of a phase means that all the planned meetings were held. Each meeting lasted 15 min, and input and output were composed of artifacts that supported the evolution of each meeting. In the initial phase, a meeting was held with everyone with the cards for the survey of the requirements and the construction of the 3D graph to represent the size. In IT meetings, artifacts, forms and tables were used to define the first packages. In the analysis and planning phases, the objectives by key results form were used. In the negotiation, we use the structural sets form. In the control phase, we have the configuration artifact and its control graph. Finally, in intelligent decision support, we use the essential questions form. The method serves as a guide for building intelligent decision support systems that can help with problems like determining whether or not to sign a contract.

• In the initial phase, cards for requirement gathering together with a complexity graph and Board Requirements by Layers and Key Person supported the organization of development packages.
• In the control phase, the input structures enabled the creation of a continuous control artifact. Furthermore, the control chart showed what is in scope and is part of ongoing control.
• The intelligent decision support phase guaranteed the refinement of requirements, which brought intelligence criteria to the development packages and gave them their unique characteristics.

## Graphical abstract

**Figure:**
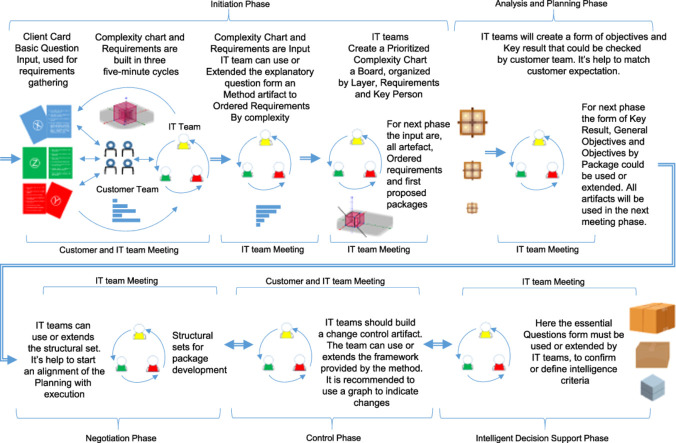

Specifications table

**Table:** 

Subject area:	Computer Science
More specific subject area:	Software Engineering
Name of your method:	Agile Meeting Method
Name and reference of original method:	Does not apply.
Resource availability:	This method can be used with any business process; in this sense, it is advisable that the client area send at least a summary of the business process and what is expected with the development of the system. From this point forward, the IT team must follow the method as described in the article using the requirements elicitation cards, the complexity 3D graph, the model and the framework artifacts. Upon request, more details on the use of the method in a specific scenario can be made available.

## Method details

This paper presents the method in detail through the Introduction, Start of Project, Initiation Phase, Analysis and Planning Phase, Negotiation Phase, Control Phase, Intelligent Decision Support Phase and Conclusion.

## Introduction

The method emphasizes the pro-active roles of the Leader and the IT team, as it was defined that the leader provides the initial treatment following the arrival of the development demand. The leader received the initial information about an incoming demand for development and made the necessary criticisms and initial considerations. It enables the existence of systems and paths that can meet the demand solution based on the initial request made by the client area that informed the title, objective, assumptions, the summary of the business process and the description of the need. For the development of this method, general issues of software engineering [1] were observed as well as specificities of agile methods [2], [3], [4] to support the development of intelligent decision support systems. As an example, an intelligent decision support system was used to help decide whether a product import contract (e.g., gasoline) should be signed at that time or not. In this sense, the IT leader compiled the initial information into something more technical for his team in order to facilitate the alignment of what was initially wanted with the demand and the best possible technical path.

In this sequence, the method takes us to the initiation phase, where we had a meeting with all those involved, and two internal ones, where the software engineers matured the business process using cards with questions or built questions that were judged to give more support to the research process. Like the already-known agile methods meetings [5,6], the method recommends that meetings last 15 min. At this stage (initial), the IT leader conducted the meeting with all those involved, separating them into groups to collect the requirements with the cards. The IT leader organized the groups, always keeping an IT team in the group. The IT team member supported the flow of the meeting and the process of identifying requirements and building the complexity 3D graph. The other two meetings in the initial phase were held only with the IT team; in these two meetings, the IT leader led the meeting in order to guide the team in organizing the requirements and separating the packages. With this, the requirements for building an intelligent decision support system were consistently identified. For example, the IT team identified the type of import it needed to use for the requested business process. In addition, the team identified variables that require access to the stock market for a more accurate decision support component.

In the meetings within the phases, input and output artifacts were used, similar to what happens in the PMBoK process groups [7]. These artifacts were used to facilitate requirements evolution and package definition. At the end of the initial phase, a complexity chart was generated, as well as a requirements board by layers and key person similar to the Kanban board [8], but with a different objective, so these artifacts were used to support the planning and definition of the initial packages. The board will mostly assist in prioritizing the packages that were prepared, as things with a key person were prioritized in order to increase trust in the development team to begin with them since they knew they would have someone to turn to.

In the analysis and planning phase, a meeting was held with everyone involved and another with the IT team. In the meeting with all those involved, the IT leader needed to ensure that his team's doubts were clarified and, at the same time, that the needs of the client area weremet. For this, the IT team leader used as main tools the board of requirements by layers and key people and the 3D complexity graph. With these artifacts, the IT leader and his team ensured the validation of the initial packages. These packages can be, for example, a package for creating an ecosystem for negotiating an import contract, or a package for connection to the stock exchange and obtaining data on the commodity. With the 3D complexity graph, it was possible to show the size of the initially thought-out system, which helped to consider deliveries of small packages. At this stage, the method expects that inputs will support the construction of general objectives, objectives per package, and key results. With these artifacts in hand, the method hopes that it will be possible to advance to a new stage.

In the negotiation phase, it was possible to more clearly define structural sets that were used in the development of the system; that is, here we can have, for example, definitions about paradigms, architecture, data integration and algorithms. At this negotiation stage, the meeting with the IT team aimed to align what was planned with possible technical solutions. For this, the IT leader used the structural set made available by the method. An example was the discussion about the paradigm that best suited the development of the negotiation ecosystem. Here, the IT leader led the meeting and participated in the discussions when appropriate, leading to reflection on whether or not to represent real-world behaviors in the trading ecosystem [9,10].

In the control phase, it was possible to define a configuration artifact so that the control was carried out continuously. A control count chart was used as support for the visualization of which types of requirements were adjusted in a given iteration. At this step, the IT lead steered the meeting into a discussion of the configuration artifact and the settings control count graph. In our example, we can cite an extension point for setting up the technical analysis, where it was possible to consider, in addition to crossing moving averages, the search for the value of the traded commodity to perform a Fibonacci expansion analysis. The IT leader was responsible for maintaining the coherence of what was being proposed. In this way, what was intended at this stage is that the continuous control [11] is always aligned with the needs of the business and always guarantees intelligent support for the decision.

Finally, in the intelligent decision support phase, the method provided questions were applied about intelligence criteria to carry out checks such as, for example, whether the framework assembled so far allows the system to identify a relevant problem in decision-making and present possible solutions. To achieve the objective in this last phase, the IT leader organized the meeting with his team in order to verify if what was planned and developed meets the intelligence criteria through the essential questions form [12,13]. In our example, we checked whether the set of algorithms was capable of identifying and solving the problem. It was possible to verify that the decision support problem for choosing the best moment to sign a contract was solved with the set of algorithms raised with the method. Thus, it was possible to define that decision support would be performed by a rule with technical bias algorithms for economic analysis or social bias algorithms with web search trends and interests, or both, to solve this problem more intelligently [14].

### Start of project

The demand that reached the project team for its development was the starting point. Thus, the INPUT was the demand with a title, objective, description, assumptions and summary of what is desired with the solution in the scope of the business process with the solution (all inputs were sent only from the perspective of the requesting area), indicating the representatives of the client area.

**Fig. 1:**
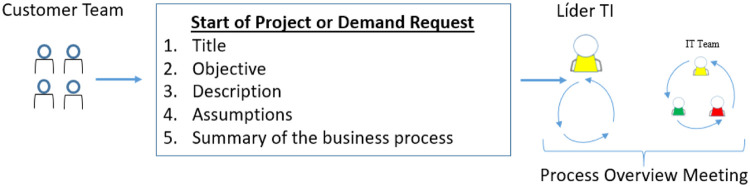
Basic data to start the project.

As an OUTPUT, the IT team leader analyzed and processed the information provided by the client area. The treatment of the information was done in order to bring the business process data into a language closer to software engineering, thus improving the understanding of the technical team in addition to proposing an initial technical path for the development of the solution. Once that was completed, the leader aligned the knowledge with the team. The dynamic described is represented by Fig. 1.

Another possibility was if the team leader understood that the request had already been answered through other existing systems and therefore suggested cancellation.

### Initiation phase

In the initiation phase, we worked with inputs and outputs, both in the first meeting that was held with the client area and in the other two meetings that were held internally with the IT team. In this phase, the meetings lasted 15 min. The method suggests that for each activity carried out in the meeting, a brief reflection of a maximum of five minutes is carried out, and this time should not be counted in the total time of the meeting.

**Fig. 2:**
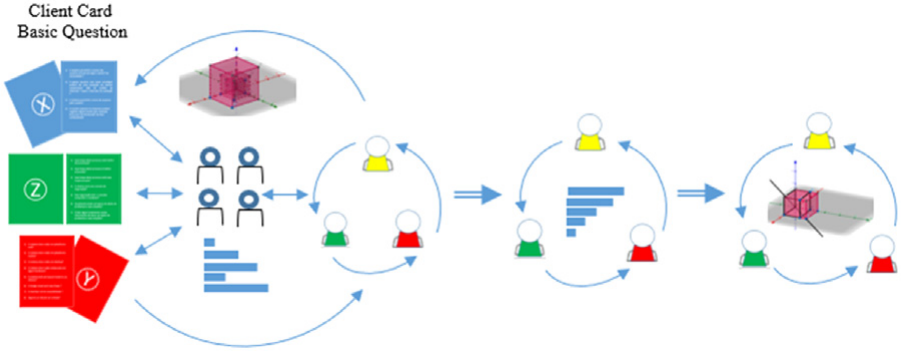
Initiation phase and its artifacts.

The method does not suggest a number of people for the meeting but only indicates that in the first meeting, the guidelines on the dynamics of gathering requirements through cards of basic questions for the customer can be done centrally or decentrally. In the centralized form, the leader of the IT area conducted the entire meeting, with all the orientation starting from him, whereas in the decentralized form, the members of the IT team play this role in a distributed manner. The INPUT and OUTPUT artifacts of the initiation phase are represented by Fig. 2.

In the first meeting of the initiation phase, cards with basic customer questions addressed to people in the requesting area were used as INPUT. The discussion based on these questions supported the requirements’ elicitation. The dynamics of the letters must be in charge of the IT area, either centrally through the leader or decentralized through the members of the IT team, so that the meeting is more fluid, resulting in a good evolution of the requirements gathering.

As an OUTPUT of this meeting, the first requirements necessary for development, were defined as a 3D graph indicating the complexity of the requested system.

During the meeting, the IT team leader kept track of the total meeting time, which lasted a maximum of 15 min. In addition, the leader controlled the time for the brainstorming activity with the cards, which is five minutes, and also controlled the time for reflection after the brainstorming with the cards; this activity must also last a maximum of five minutes, which will not be counted in the total meeting time.

The method made the cards with basic questions available to the client, presenting ready-made questions that support and direct the beginning of the requirements survey, but in addition to having these questions, the method leaves software engineers free to create their own questions, and so the cards work as a framework.

It was in this reflection activity that the IT leader needed to organize the requirements on their respective axes and began to draw the complexity chart. The 3D graph has the number for requirements of each type, functional, non-functional, and intelligence or behavioral, on its axes, so that every five minutes a drawing was defined. This graphic tends to grow every five minutes, and each story is recorded in the drawing. By the end of the meeting, the method recommended making three complexity charts so that the client area could visualize the evolution of the complexity and size of the desired software.

X Axis: Functional Requirements

Y Axis: Non-Functional Requirements

Z Axis: Intelligence or Behavioral Requirements

Next, let's see the cards with basic questions for the client and then the complexity graph that is the result of the activity with the cards.

### *Cards with basic questions for the client*

The cards directed to customers contain the previously established requirements and axes, thus enabling the construction of a complexity graph; for its part, this graph is the result of the task performed with the cards. Thus, the method defined that the X axis deals with the Functional Requirements and, hence, the forms that directed questions for the survey of these requirements.

**Fig. 3:**
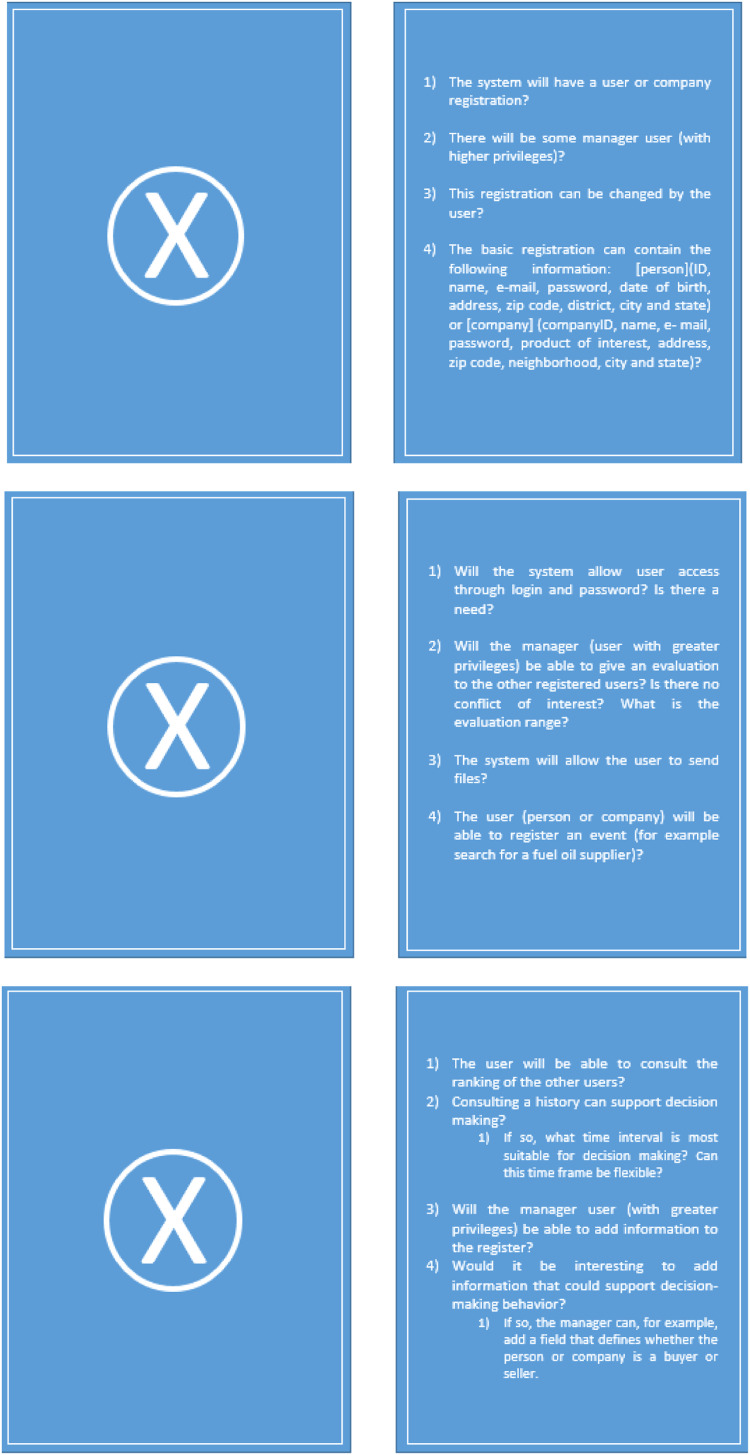
Three X axis cards, to the client to gather functional requirements.

These are the blue cards that have the letter X on one side and the questions that drove the customer survey on the other side. The method brings ready-made questions to these cards but also allows the IT team to modify or add new questions, providing the flexibility typical of the framework. Below are three initial cards made available by the method for collecting functional requirements (Fig. 3).

**Fig. 4:**
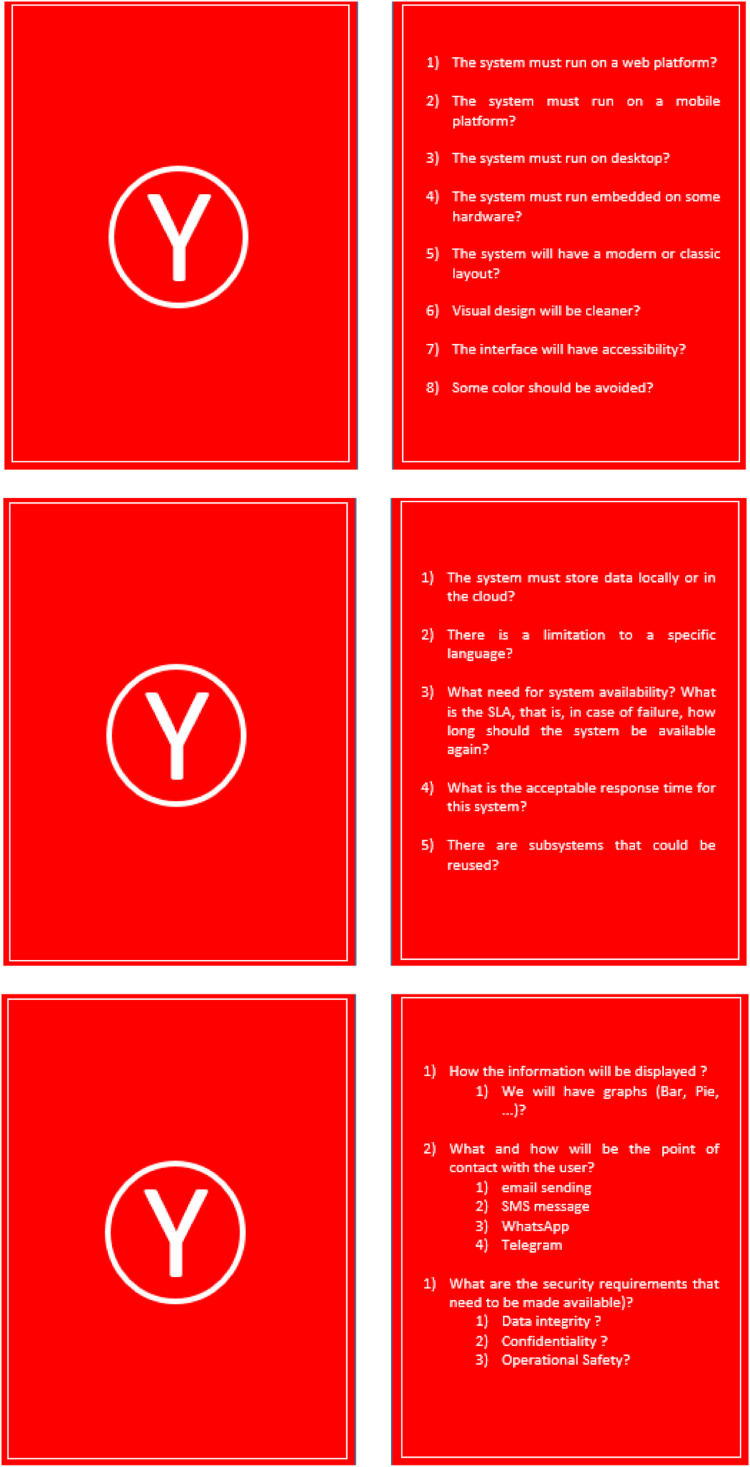
Three X axis cards, to the client to gather non-functional requirements.

The red cards have the letter Y on one side and the questions that drove the survey with the customer about non-functional requirements, as defined by the method, on the other. On this axis, the method also introduces ready-made questions for these cards and, furthermore, allows the IT team to change or add new questions. Below are the three initial cards made available by the method for collecting non-functional requirements (Fig. 4).

**Fig. 5:**
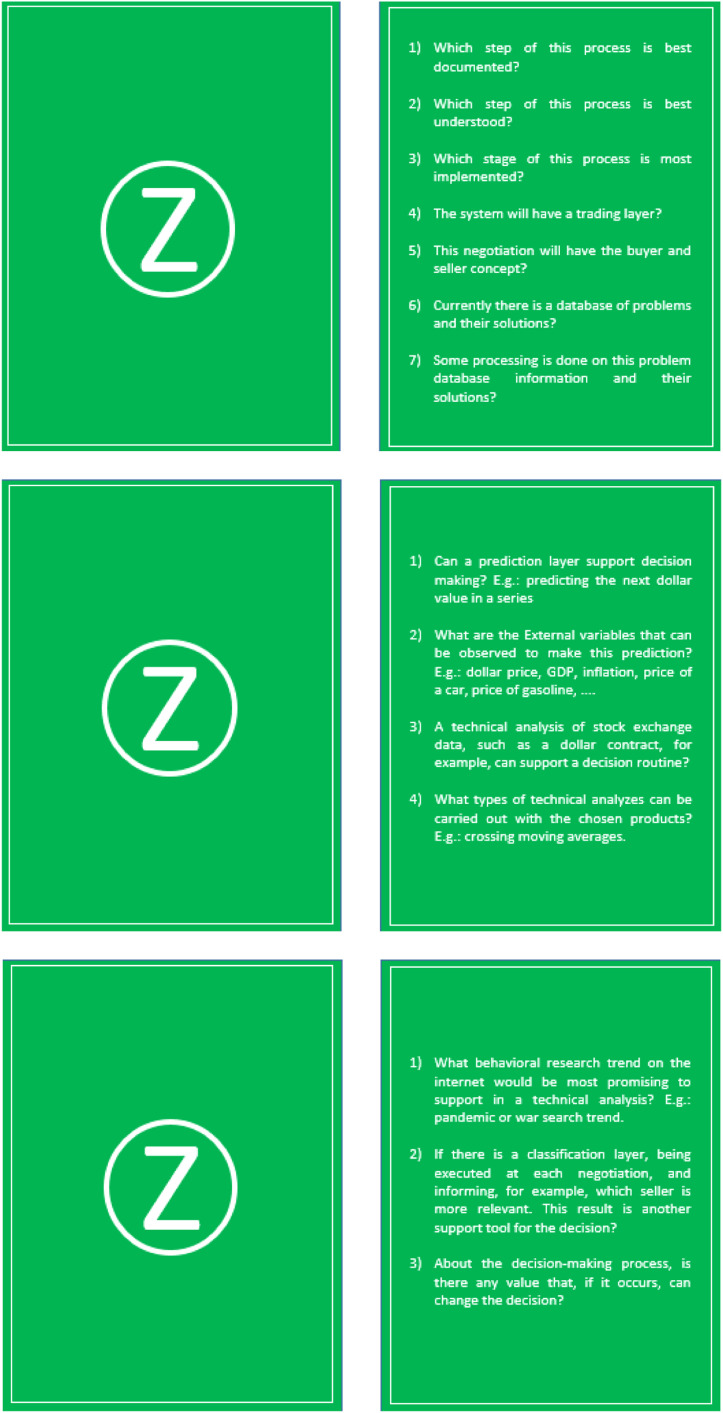
Three X axis cards, to the client to gather intelligence or behavioral requirements.

With the green cards, we defined the Z axis, where the method provides questions that supported and streamlined the research with regard to intelligence and behavior requirements. On this axis, the method both provides ready-made questions for these cards and also allows the IT team to change or add new questions. Below are the three initial cards made available by the non-functional requirements collection method (Fig. 5).

**Fig. 6:**
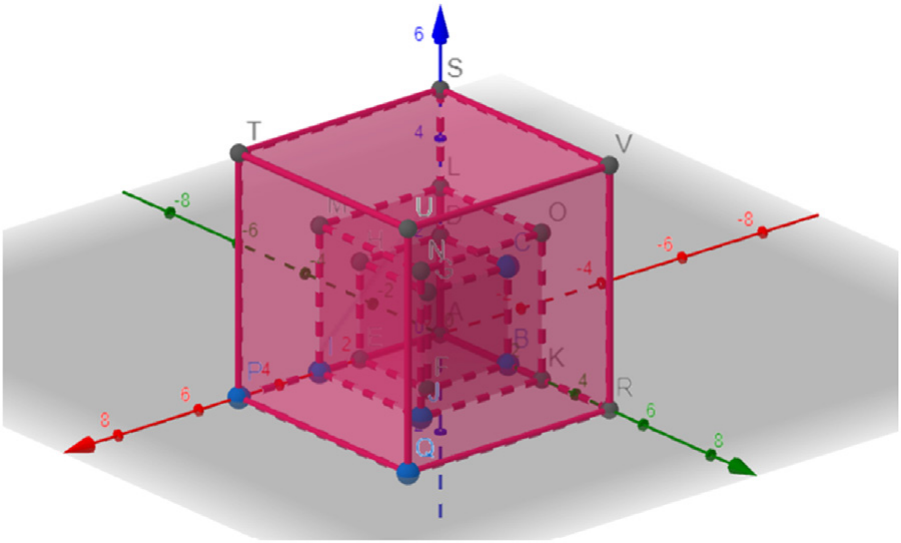
Complexity 3D graph.

As defined by the method, after every five minutes of surveying with the cards, we build a complexity graph. To produce this drawing, the method allows two possibilities: one is to draw on paper, while the other is to draw using geometry software, which can be found on the web, for example, at geogebra (https://www.geogebra.org/3d), which is where we created the 3D graphics that are in this article (Fig. 6).

The graphic tool offers several advantages; let's highlight some. One of them is the possibility of quickly presenting the evolution of the size and complexity of the requested software to the client area. Furthermore, with the graphical tool, it was possible to show more clearly the advantages of an evolutionary development trajectory with small deliveries. From an IT perspective, the graphical tool provided greater clarity on the division of tasks and package complexity.

It is possible to visualize and present the complexity of the packages that were developed in a simple and efficient way through this graph, allowing for more agile decision-making.

In the initial phase, in the meeting with all those involved, the survey of requirements was carried out, and then a graph was drawn with the number of requirements, each on its respective axis. In this way, at the end of each survey time, the IT team constructed the complexity 3D graph; this step is not included in the meeting time. The meeting can have three rounds of discussion for requirement gathering, where each round counts for five minutes, and after that time, the construction of the complexity chart begins. In this process, the cards were an acceleration artifact that could make the process more dynamic and fluid.

This method has the benefit of agility by graphically presenting the complexity of the software that was requested. This representation is an advantage compared to traditional methods as a function point, which demand greater efforts and sometimes do not present the complexity of the system to the user in such a direct manner. Already in the first meeting, the method permitted a rapid analysis of what is to be developed, enabling an examination of the packages.

Also in the initiation phase, the method recommended holding two more meetings in which only the IT team participated, each lasting 15 min. The objective was to organize the requirements and compose smaller packages aimed at future deliveries.

As INPUT for this first meeting with the IT team, the 3D graph of the system's complexity and the requirements raised in the previous meeting with the client area are expected. As an OUTPUT, the method expects the requirements to be realized to be ordered; the ordering followed the criterion of greater development complexity. In this sense, another artifact that can be used or expanded by the IT team is the explanatory question form, which aims to improve the understanding of software engineers about the business process as well as the techniques and strategies used by the user in this business process (Table 1).

**Table 1:** Explanatory question form.

Explanatory Question Form	
i) 1. How does the Negotiation Process work? 2. What is considered when making the decision? 3. How to improve the decision-making process? 4. How to streamline the decision-making process? 5. What are the main difficulties in decision-making?	ii) 1. What level of decision-making should the system handle? 1. Strategic Decision 2. Tactical Decision 3. Operational Decision 2. How is currently existing decision-making classified, programmed or non-programmed (or both)?
iii) 1. Is there any support tool for this decision-making? 2. What are the main logistical problems of this process? 3. Do negotiators receive support for their decisions? 4. What is the scope of this process?	iv) 1. How would you rate the decision according to probability? 1. Risk decision? 2. Decision uncertainty? 3. Sure decision? 2. How would this decision be classified according to the deadline? 1. Short-Term Decision? 2. Long-Term Decision?

This artifact can be used or extended like a framework.

The basis for preparing the Explanatory questions was the experience in developing systems for decision support in the business and industrial scopes, where systems aimed at the industrial scope were developed for the oil industry and business decision systems were developed for a pension and consulting company in the field of informatics. The experience of the requirements survey meetings for the construction of these systems showed that some questions accelerate and boost the survey and understanding of the system that is intended to be developed. And the consolidation of this type of structure as an artifact to be used in the method occurred through articles [12,13]. These studies reinforced the understanding that questions can accelerate the feedback process in requirements gathering, so we believe they represent a good tool for the initial phase of the method. We understand that in some scenarios the number of questions may be sufficient and in others not; therefore, the method understands that these explanatory questions can be seen as a framework, that is, can be used as provided or extended by the user so that he can create questions or just complement the existing questions, thus adapting to his scenario when convenient. The other artifacts of the method follow this form of use, i.e., they can be used as a framework or as a template.

This form is made available by the method, so the pre-filled questions can be used by the team, or the IT team can modify them or create new ones. If the team wants to create new questions, the method indicates creating questions similar to the 5W2H standard for building questions, i.e., WHO, WHEN, WHERE, WHY, HOW, WHAT, and HOW MUCH [3,8].

From that point on, some questions such as, for example, “What is the problem to be solved?” and “Why do you need this system?” have already been answered, so it is expected that other questions such as, for example, “Where does the current system run?” “Who is responsible for performing each action?” “What is the frequency of carrying out this action?” “How can we improve the current form?” and “What is the cost involved in carrying out the process?” must be raised by the IT team.

In turn, the IT team leader must keep in mind a concept that is more closely linked to the action plan [16] in order to support the team on the way to achieving the objective. In this way, he was responsible for compiling and relating the questions to an action plan; that is, he must carry out an iterative process, always bearing in mind that the questions referring to “what” brought the actions, the steps; questions referring to “why” brought justifications and reasons; questions referring to “where” brought location within the system architecture; questions referring to “when” brought dates, deadlines and periodicities; questions referring to “who” should bring those responsible for the action; questions referring to “how” presented methods and processes; and, finally, questions referring to “how much” brought cost data to the solution. In the proposed method, we do not deal with the scope of cost in depth; we only brought a view of complexity from the 3D graph and that increasing this complexity increases cost and difficulty.

An iterative cycle was used to populate this form, which was then distributed among the IT teams. The method uses three cycles of five minutes. In the first cycle, the software engineers had five minutes to modify or build the questions in a certain space on the form, and in the second meeting cycle, they exchanged forms with colleagues; this exchange can be random or by a rule that can be defined by the leader during the meeting. This second cycle is carried out so that the software engineers can, based on the questions, analyze and also reflect on the vision of the other team members. In the last cycle, the leader will present the answers to all the questions created for the team. At this stage, the team must, through consensus, choose the questions that most help in the business process and in the development of the intelligent decision support system.

The method advocates that, from the beginning of the meetings, it is necessary to keep in mind the evolutionary concept of understanding the business process. It is always necessary to understand if the raised requirements and the proposed architecture are aligned with the solution design and if the algorithm used for intelligence meets the requirements.

From this point on, we can move on to the second meeting, where the method expects the INPUT artifacts to be the requirements raised with the client area, organized in ascending order by complexity, in addition to the answers in the form of explanatory questions. As an OUTPUT, the method expects a framework to be built, arranging the requirements by layers and key persons — people who can support the development. This layered form of requirements and key person supported the resizing of the 3D graph of system complexity; that is, the team prioritized packages based on the artifacts so that a new 3D graph is drawn.

In this meeting, the team used the framework concept similar to that used in the Kanban board [15], but one of the columns of the framework should be considered the layer of a real system. The method defines the use of the negotiation layer, the data layer, and the prediction and splitting layer. Finally, it suggests a last layer, such as UX/UI or others. The team can complete the table in two ways: one of the ways is to place the requirement in a more appropriate layer; another is for the team to create technical actions that represent one or more requirements and thus place this action in the corresponding layer, always indicating the key person. So, we had the filling of the layers, the requirements, and the key person chart. This board-filling process can be achieved with Post-it notes on a whiteboard or digitally (Table 2).

**Table 2:** Requirements chart by layers and key person (used technical actions).

Boarder by Layer, Requirement and Key Person			
negotiation layer	data layer	prediction, rank layer	ux/ui
real simulation	stock market	pagerank	best color
user contact telegram	google trend	lstm	usability
sensor definition	data lake	k-nn	layout
key person	key person	key person	key person
Maycon	Jhon	Will	???

This artifact could be used like a template.

**Fig. 7:**
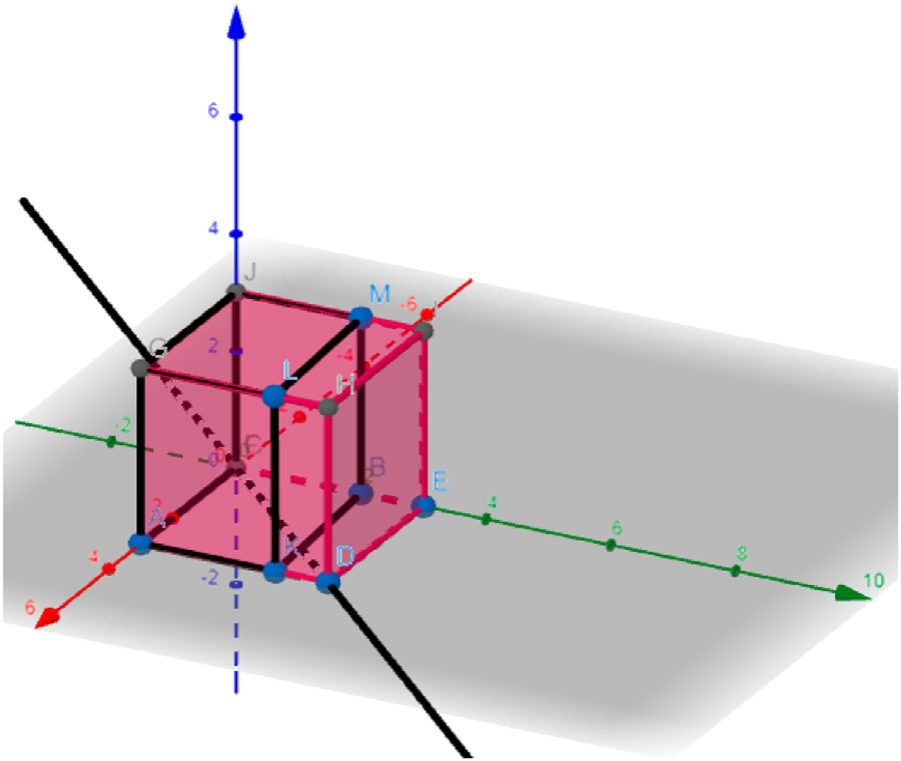
Prioritized 3D graph.

In this meeting, the team should also indicate focal points for each layer of the frame. With this mechanism, the method hopes to provide greater support to the team in prioritizing packages as it becomes visible if there is someone with the necessary knowledge to handle the demand. In this way, the framework brought more confidence to the team and greater support for prioritization. Thus, the prioritized 3D graph was supported by a board of requirements, layers, and key person (Fig. 7).

In the last meeting of the initial phase, the IT team and its leader got together to consolidate their understanding and define a framework of requirements, by layer and by key person, which is the artifact that supported the definition of the packages that were planned for the initial delivery. With this artifact built, we can more objectively present the size of each initial package and the complexity of each task to the client area.

After the IT meetings, the Leader is expected to carry out a critical analysis of the survey done so far; this analysis can be done alone or with his team. The method suggests that this analysis follows a conceptual iterative process where the beginning of the cycle takes place with an understanding of the business process proposed in the method in order to carry out its consideration of the evolution of requirements, planning and development. In this way, the leader must first reflect on the requirements raised for the understanding of the business process and the planning given for the architecture. In the sequence, one should reflect on the design of the solution to see if it is following the plan to meet the business process. The next step is to deepen the discussion of the algorithm for the business and its intelligence, as well as the way in which communication with the user will be carried out. Finally, the discussion should proceed to the technical definition of the implementation of the database to enable the way of negotiating the business process, in addition to moving on to the reflection of the prediction and classification of the information that will support decision-making.

The method makes it possible to conduct the initiation phase cycle once again, with yet another meeting with the client area and also the two internal meetings of the IT team. Therefore, the method ensures that everyone involved has a greater understanding of the process and what is to be constructed. Finally, the method recognizes that in this second cycle, the meetings will be more productive as they take place with several artifacts in hand.

### Analysis and planning phase

**Fig. 8:**
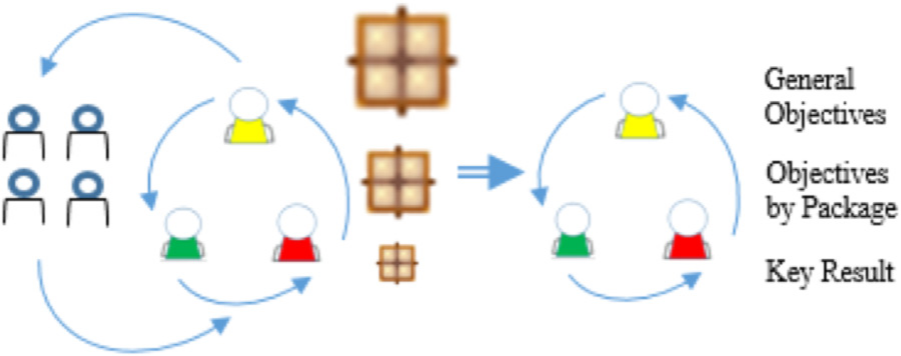
Analysis and planning phase.

In the analysis and planning phase, we have a flow similar to that performed in the initiation phase, where we had a meeting with everyone involved and another with the IT team. A highlight of this phase is OUTPUT, in which the general objectives are designed by packages and key result, which also leads us to a confirmation of the definition of the scope that will be effectively treated. The INPUT and OUTPUT artifacts of the analysis and planning phase are represented by Fig. 8.

As INPUTS, we had all the artifacts that were concluded in the meetings of the previous phases. The meeting with all those involved was held based on these artifacts and should aim to obtain approval from the client area on the initial definitions of the packages. The board of requirements by layer and key person was used as support in the discussions, as can the other artifacts, and this allows us to analyze all definitions.

In the meeting with the IT team, we had all the previous artifacts as an INPUT, but now with the approval of the packages by the client area; and as an OUTPUT, we had the planning for the deliveries of these packages, thus enriching the board of layer by requirements and key people, where details such as the technology to be used and architectures to be built for each task must be entered. From this meeting, where the data is enriched, we can then build the objectives by packages and in general.

To support this meeting and the process of constructing the objectives, the method incorporates the table of objectives into the primary outcome. This artifact is one of the frameworks we employ, which in this case already presents general objectives and objectives by packages as a means to achieving the general objective and key result that can be used and verified. But this table should be used as a framework [17,18] in which software engineers can modify or create new sentences (Table 3).

**Table 3:** Objectives by means to achieve and key results form.

General Objectives	Means to Achieve	Key Result
Perform Contracts with Less Risk	Implement Sensors to obtain information that can be treated in order to reduce risks. Enrich data to get a better result in a next iteration.	Obtain real-time quotes for currencies used in international contracts. Having a history of information that makes it possible to minimize risks in decision-making.
Make the company relevant in the market	Implement information prediction algorithms. Implement information classification algorithms. Implement supplier ranking algorithm.	Increase the percentage of contracts with a positive result in the company's sales. Create supplier registration by ranking.
Implement criteria that anticipate risk	Implement technical analysis of the stock exchange. Implement interest-based social analytics of web searches.	Improve anticipation of crisis scenarios. Avoid entering into contracts during these periods.

This artifact can be used or extended like a framework.

With this table, we can also see that the means to achieve the general objectives will be the objectives of the packages, which confirms all the planning to be carried out.

### Negotiation phase

After the negotiation phase, the method enters a stage of the cycle in which it is not possible to return to previous phases. In the negotiation phase, the IT team must work harder to define the strategies, algorithms and architectures that will make the presented business rule viable. The objective was to delve into conceptual issues in order to align the planned solution with what will be developed, so we will have, for example, the alignment of the planned architecture with the technology that will be used to make this solution viable. This alignment strategy must be implemented at each method execution cycle so that, in each phase, it is possible to prioritize the approach that can construct the most intelligent system.

In this phase, the INPUTS were comprised of all the outputs of the previous phases, so all the enrichment of information that was generated in the previous phases can contribute to the analyses and weighting necessary to define the structural sets, which are divided into three groups: architecture, paradigm and forms of integration. To speed up this process, the method provides a list of questions with options for each structural set of definitions of program structures. As with other artifacts, it can be used as is or extended to include a set that the team understands to be more relevant (Table 4).

**Table 4:** Structural sets form.

Structural Sets	
Set of main paradigms	Which paradigm is best suited for system development? Is one of them enough to serve the entire system or will it be necessary to use different paradigms in each component? Object Oriented Paradigm Event Oriented Paradigm Agent Orientation Paradigm
Set of main Architectures (architectural styles)	Which architecture and architectural style is best suited for the system? Layered Architecture Client-Server Architecture MVC Architecture Service Oriented Architecture SOA Micro Services Architecture
Set of main forms of Integration	Which integrations currently exist, which need to be created, and which type of integration would be most suitable to obtain all the necessary information for an intelligent decision support? 1. Integration via APIs. a. Is there already a system that will support the development of this new demand and that uses this type of integration? 2. Integration via webservices (e.g., SOAP and REST that can use standard XML, JSON, HTTP …). a. Is there already a system that will support the development of this new demand and that uses this type of integration? 3. Integration vsia messaging via .CSV, .txt, … a. Is there already a system that will support the development of this new demand and that uses this type of integration? 4. Integration through Middleware a. Is there already a system that will support the development of this new demand and that uses this type of integration? 5. Data integration through ETL (Extract, Transform, Load) used to integrate data from different sources (database or files, …). a. What will be the data sources? (Structured, semi-structured and unstructured data) Database? XML files? Text files (.csv, .txt, …)? b. How many databases will be used? i. We already have access and users in this database c. What are the repositories for the XML files? i. Is there an API to extract this data? (If so, seek access to these APIs. If not, understand and build RPA for data integration and extraction). d. What are the repositories for .CSV files? i. Is there an API to extract this data? (If so, seek access to these APIs. If not, understand and build RPA for data integration and extraction) Is there important data that is not in any repository or database? Is it online data? Is there a form of connection/integration?

This artifact can be used or extended like a framework.

The OUTPUT artifact was a structural set that was chosen through the consensus of the IT team involved; this set was used to carry out the development of the packages that were defined and must indicate important points; for example, the paradigm that was used to carry out a simulation of real negotiation [9,10] and the paradigm used to develop the other business rules. These definitions can be made based on the layers suggested by the method in the analysis and planning phases. This definition process was guided by the leader and was under the responsibility of the leader and his team. The team should discuss the entire list of structural sets presented during IT meetings, and existing or new questions should include questions, such as, “Which paradigm makes it possible to get closer to a genuine negotiation?”

### Control phase

At this stage, there is a small meeting with the client area to align management and planning, as well as obtain the approval of the packages being developed.

The INPUT artifacts were all the outputs that were generated in the meetings of the previous phases. This process of increasing the input artifact allows us to always keep alive the memory regarding the business process and the decisions we make, and this supports the construction of a more assertive control.

Control is fundamental so that we can understand whether something is approaching its objectives, and to ensure that planning is not going off the rails, we must plan the adjustments that will guarantee control. Adjustments can occur when we have positive or negative deviations; positive deviations occur when the result goes according to plan but can still improve; that is, in this case, we have an opportunity for improvement to take even more advantage of the system. Negative deviations, on the other hand, occur when the result obtained is lower than expected, and therefore corrections must be made.

The control helped us to reassess the practices used in development, in addition to the manner of negotiating and the decision-making methods used.

As an OUTPUT, the control phase generated a configuration artifact, which in practical terms we can imagine as being a configuration table, so that the whole system can use it in order to adapt to the “new” practices and rules, thus following the needs of the area and of the business. The result was that the configurations made in this artifact will always pursue the most appropriate way of making an intelligent decision and, consequently, the achievement of the established objective.

The configuration artifact was able to add controls to the system in order to allow adequate direction of the environmental variables, making it possible to reach the defined objectives. In this way, the configuration artifact was a mechanism that allowed the system user to add, for example, technical analyses for the treatment of an asset on the stock exchange. So, if the system is performing an analysis based on moving averages, the artifact must allow the user to add or change an analysis. With this, it was possible to add a Fibonacci expansion analysis if it is understood that, for this scenario, this analysis can replace the process for achieving the goals. The method provides a configuration table with some control points (Table 5).

**Table 5:** Configuration artifact form.

ID	Type	To do	Extension Point on code
X0001	Char	Include var gas to add new interest (for oil contract)	Main definition of interest
Z0001	Function	Include function getValue() of Brent on stockMarket	Main function of stockMarket values
Y0001	Char	Include topColor new color on top of page	Main page layout
Z0002	Function	Include function fibonacciExpansion on stock exchange assets	Main function of stockMarket prediction

This artifact can be used or extended like a framework.

In this configuration table presented by the method, we see the ID making a correlation with the requirements axes (x,y,z) presented in the 3D complexity graph, and here in this structure, we have the places where these control points can be inserted. The structure also presents what should be implemented, the type of data, and which requirement it was impact.

**Fig. 9:**
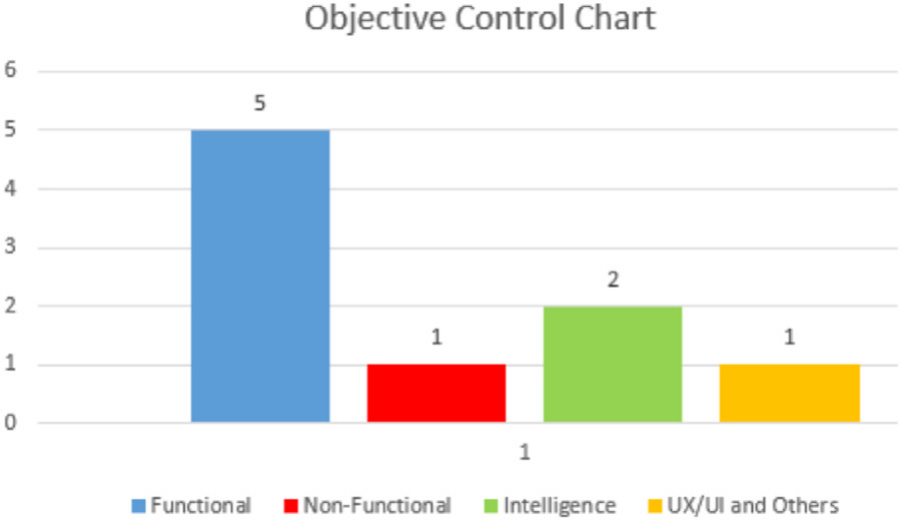
Objective control chart.

Finally, to measure how much control we have, the method suggests using a bar chart to track objectives since it is a simple artifact and will serve as support for managing the controls that are being created in the configuration table. With it, it was possible to see the number of requirements that this possibility of adjusting the trajectory has to meet in order to reach the goal (Fig. 9).

In the bar chart, the number of controls mapped to each axis was marked; these controls can include planned and implemented controls.

### Intelligent decision support phase

In this phase, the IT team will focus on identifying criteria that enable intelligence. For this, the method provides a form with some essential questions that must be asked in meetings. As with the other artifacts, this form can be extended, and the team can modify existing questions as well as create new ones. At this stage, it is expected that the planning and/or construction of an intelligent and self-adaptive architecture [19] will be carried out, and, to this end, the method advocates the use of frameworks and paradigms that enable better management of intelligence and behavioral issues.

The INPUTS for this phase were all the outputs of the previous phases, thus bringing a large volume of organized and enriched data to the discussion, which enables an analysis of the process at a deeper level.

As an OUTPUT of this discussion, we determined whether the assembled structure is sufficient to give the system a behavior that meets intelligence criteria or whether it is necessary to add additional conditions for the system to reach the desired level of maturity (Table 6).

**Table 6:** Essential questions form (Intelligence Criteria).

Essential Questions (Intelligence Criteria)
(1) With the assembled framework so far, what is the potential of the system to identify relevant problems in decision-making? (a) Do the identified sensors provide enough information for the control system to detect these problems?
(2) Is there a set of skills/algorithms in the software capable of solving this problem?
(3) Is there an evolutionary history of data supporting intelligent decision-making?

This artifact can be used or extended like a framework.

At the end of this meeting, it is expected that the planning of the packages and the conditions for the intelligence of the system will be specified. In this way, the IT leader can now return to iterating between the phases of the second stage of the method, that is, the negotiation phase, the control phase, and the intelligent decision support phase.

The method expects that everything requested is planned and designed in the first pass through all phases, although it is possible to start package development along with this first conceptual cycle. In this sense, it is expected that the conclusion of the development of all packages be completed as of the next iterations between the phases of the second stage of the method, and thus the deliveries [20] were planned and developed in a more assertive manner.

**Fig. 10:**
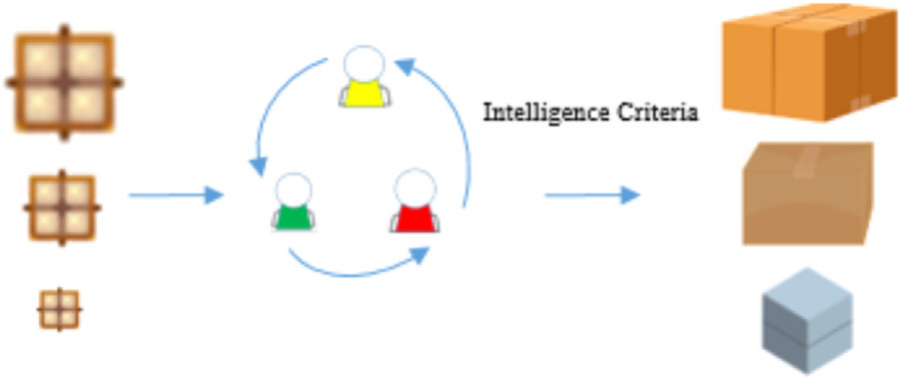
Intelligent decision support phase.

Fig. 10 shows that the initial packages come with a certain level of intelligence assessment, and after the intelligent support phase, the decision comes out with an even more elaborate one.

## Conclusion

The agile meetings process aims to support the framework for building an intelligent decision support system, but it is not limited to just this type of system. In this sense, the cards with basic questions for the customer are hotspots of the framework, allowing software engineers to extend these points when necessary. In the same way, the other forms introduce the possibility of performing the extension by modifying or including more questions so that all those more pertinent to the process and the rules involved are clarified.

At the end of several cycles of the method, we will have completely reusable outputs as a result, along with an inclination to build intelligent software.

With this method, we can not only carry out a survey in an agile manner but also build an intelligent plan where we can identify inconsistencies, disagreements and obscure aspects in advance.

Finally, the method allows for the planning ofa structure that aims to deal with the variability that exists in the execution environment of the process so that it is possible to optimize both the process and the decision-making to achieve the defined objective. We understand that the method allows us to present elements that can complement the cognitive process inherent in the construction of an intelligent decision support system.

## Ethics statements

Does not apply

## CRediT authorship contribution statement

**Guilherme Nascimento Pate Santos:** Conceptualization, Methodology, Software, Formal analysis, Investigation, Data curation, Writing – original draft, Writing – review & editing, Visualization. **Carlos José Pereira de Lucena:** Investigation, Resources, Writing – review & editing, Supervision, Project administration, Funding acquisition.

## Declaration of Competing Interest

The authors declare that they have no known competing financial interests or personal relationships that could have appeared to influence the work reported in this paper.

## Data Availability

Data will be made available on request.
